# Production of Chemically Modified Bio-Based Wood Adhesive from *Camote* and Cassava Peels

**DOI:** 10.3390/polym16040523

**Published:** 2024-02-15

**Authors:** Anna Mae Rabaca Reotutar, Roselle Yago Mamuad, Angelo Earvin Sy Choi

**Affiliations:** 1 Department of Chemical Engineering, Mariano Marcos State University, City of Batac 2906, Philippines; annamaereotutar@gmail.com (A.M.R.R.); roselle_mamuad@dlsu.edu.ph (R.Y.M.); 2 Department of Chemical Engineering, De La Salle University, 2401 Taft Ave., Manila 0922, Philippines

**Keywords:** bio-based adhesives, *camote* peels, cassava peels, renewable, sustainable

## Abstract

Adhesives are significant for manufacturing competent, light, and sturdy goods in various industries. Adhesives are an important part of the modern manufacturing landscape because of their versatility, cost-effectiveness, and ability to enhance product performance. Formaldehyde and polymeric diphenylmethane diisocyanate (PMDI) are conventional adhesives utilized in wood applications and have been classified as carcinogenic, toxic, and unsustainable. Given the adverse environmental and health effects associated with synthetic adhesives, there is a growing research interest aimed at developing environmentally friendly bio-based wood adhesives derived from renewable resources. This study aimed to extract starch from *camote* and cassava peels and focuses on the oxidization of starch derived from *camote* and cassava peels using sodium hypochlorite to create bio-based adhesives. The mean yield of starch extracted from *camote* and cassava peels was 13.19 ± 0.48% and 18.92 ± 0.15%, respectively, while the mean weight of the oxidized starches was 34.80 g and 45.34 g for camote and cassava, respectively. Various starch ratios sourced from *camote* and cassava peels were examined in the production of bio-based adhesives. The results indicate that the 40:60 *camote* to cassava ratio yielded the highest solid content, while the 80:20 ratio resulted in the best viscosity. Furthermore, the 40:60 ratio produced the most favorable particle board in terms of mechanical properties, density, thickness, swelling, and water absorption. Consequently, the starch extracted from *camote* and cassava peels holds promise as a potential source for bio-based adhesives following appropriate chemical modification.

## 1. Introduction

The significant role of wood adhesive in the progress and success of the woodwork industry has been recognized recently. The wood adhesive is identified as one of the factors in the efficient usage and management of wood resources. By far, the adhesive market is known to be increasing rapidly, and it is expected to continue in the future [1]. According to Global Market Insights, the worldwide demand for adhesives was $63 B in 2022 and is forecasted to reach $102 B in 2032. Many adhesives are being used in the construction industry, particularly in those manufacturing building materials such as plywood and particle board. The largest use of adhesives is in the construction industry. [2].

Four thermosetting resins are conventionally utilized as wood adhesives, phenol-formaldehyde (PF) [3], urea-formaldehyde (UF) [4], melamine formaldehyde (MF) [5], and PMDI resin [6,7]. PF adhesives are typically formed by synthesizing phenol and formaldehyde with the aid of alkaline catalysts [8]. PF is an excellent adhesive for plywood and particle board production for building boats. PF wood adhesive is characterized by its great moisture resistance and is widely utilized in the production of oriented standard boards, softwood plywood, and siding [9]. UF adhesives are utilized in the wood industry for bonding wood and wood pieces to form useful wood products such as plywood and particle board [10]. It is usually produced by adding formaldehyde to urea under alkaline conditions and in the presence of catalysts [11]. MF adhesives are utilized most for exterior wood panels because of their excellent water resistance capabilities compared to other formaldehyde-compound adhesives [12]. MF is expensive; therefore, it is usually blended with urea to make it cheaper [13]. PMDI adhesive is characterized by its excellent bonding strength, efficient water, and climate resistance properties. Their expensive cost is usually overcome by their faster curing time, but they have no comprehensible benefits in terms of sustainability or environmentally friendly features compared to formaldehyde-compound adhesives [14]. Formaldehyde and PMDI have been categorized as carcinogenic and toxic materials based on the material safety data sheets (MSDS), and the production of these resins has relied on non-renewable petroleum resources [15]. Due to the negative impacts of synthetic adhesives, there has been growing interest in the development of bio-based adhesives that are environmentally friendly and come from renewable sources [16]. Synthetic wood adhesives are made from non-sustainable petrochemical materials. These materials, such as formaldehyde, are known to contain volatile organic compounds and residual hazardous compounds. One of the carrier fluids used in conventional heat-melt adhesives is toluene, and this solvent is quite environmentally damaging and unsafe. Several resources from plant materials have been utilized as sustainable feedstock to chemically modify bio-based adhesives. These plant materials include lignin, plant proteins, tannins, bark, starch, and vegetable oils. Numerous studies on these biomass sources have been explored to find out their potential as renewable sources. Some of these are sweet sorghum juice as a potential raw material for alcohol production [17] and peanut shell and *cornick* industry wastewater as raw materials for briquette production [18]. Optimization studies on these biomass sources have also been explored [19].

Starch is a white organic powder produced from natural carbohydrates extracted from plants. They are usually taken from the tubers, roots, and seeds of plants [2]. Starch is one of the potential raw materials for the advancement of bioadhesives. Starch as a bioadhesive feedstock possesses several advantages, such as sustainability, a cheap, simple production process, excellent bonding performance, and excellent film construction [20]. Starch contains three hydroxyl groups attached to every unit of glucose that can establish hydrogen bonding. Adhesives made from starch have been popularly utilized in wood manufacturing, but the ability to bond wood is not sufficient to meet the required strength. The starch has a strong attraction to hydrogen bonding, which causes its strong affinity for water. This characteristic causes starch-based bioadhesives to dry over time. The utilization of starch directly as bioadhesives is unsuccessful because of problems in the storage and the standard bonding strength of wood adhesives. Wood adhesives made directly from starch have poor bonding strength compared to standard. It is also characterized by poor water resistance, unlike wood adhesives containing formaldehyde. Therefore, to produce high-quality starch-based wood bioadhesives, their molecular structure should be strengthened. The inferior quality of starch as a bio-based adhesive can be enhanced through chemical modification. One method of chemical modification of starch is an oxidation process [21]. The oxidation process occurs when the hydroxyl groups are converted to carboxyl groups in the glucose part of the starch. The disruption of glucose rings resulted in carboxyl, carbonyl radicals, and depolymerization. The oxidants are commonly used in the modification of oxidized starches. Starches may be oxidized by sodium hypochlorite, bromine, potassium, hydrogen peroxide, ammonia persulfate, and potassium permanganate [22].

One of the plants that is an excellent source of starch is cassava. Cassava is a root vegetable or tuber containing a large amount of starch commonly eaten worldwide. The plant originated in South America and is known to be an efficient supplier of fiber, vitamin C, thiamin, folic acid, manganese, and potassium. Cassava is described as a long-standing plant with fan-shaped leaves that contains fleshy brown roots, or tubers. Cassava starch as a raw material for bioadhesives is more advantageous than other starches because of the clear paste formed, the low gel formation temperature, stability of the gel formed, and its excellent film properties [23]. Sweet potato, locally called *camote*, is another source of starch. It is an herbaceous perennial plant commonly known as a tribe in tropical countries. The leaves are triangle-shaped and attached along the stems. The leaf is usually 5–10 inches long, crawling on the ground. It is a root vegetable or tuber that is big, rich in starch, and usually tastes sweet. It is an underground tuber known to be full of nutrients, rich in fiber, good tasting, and usually eaten boiled, baked, steamed, and fried. This plant originated in tropical countries in the United States [24].

The use of wood adhesive that is considered eco-friendly and safe for humans will lessen the negative impacts on the environment because it will not emit strong fumes that can harm the health of an individual. The use of this kind of adhesive is important because sources will be renewable and accessible [25]. The production cost is increased, and the biodegradability of chemically modified bio-based adhesives is decreased during their production; however, their advantages with regards to petrol-based adhesives are still superior. Chemically modified bio-based adhesives are still cheaper, produce a lower carbon footprint and lower toxicity, and still possess higher biodegradability and a more sustainable design than petrol-based adhesives [26].

Extracted starches are commonly used for food and food-related purposes. Considering the utilization of food starches from food sources, this will cause disputes and rivalry among food and food-related manufacturers. Food security will also be a concern once the starch-based adhesive is fully adopted. Exploring starch-based adhesives has become a recent research interest, such as the studies on biobased adhesives formulated from tannic acid, chitosan, and shellac [27], research on starch-based adhesives for wood panels [28], improvement of starch-based adhesives with carboxylic acids and enzymatically polymerized lignosulfonates [29], and evaluation of the properties of starch-based adhesives [30], to name a few. Many studies have been conducted on the potential of starch as a bioadhesive, but the potential consequences of utilizing starch-based adhesives were poorly given attention. Exploring alternative sources of starch that would not compete with food sources would be an excellent step to adopting these innovations [31]. The study aims to develop a wood adhesive from cassava and *camote* peels. Specifically, it aimed to determine the percent yield (% *w*/*w*) of starch from *camote* and cassava peels; determine the amount of chemically modified starch produced from *camote* and cassava starch; characterize and compare the best ratio of *camote* and cassava starch as chemically modified bio-based wood adhesives (CMBWA) in terms of solid content, viscosity, and glass transition temperature (T_g_); produce particleboard using the different *camote* to cassava starch ratio for CMBWA and analyzed in terms of mechanical properties namely, compressive strength, modulus of rupture (MOR), and physical properties such as density, water absorption and thickness swelling, and determine the best proportion among the *camote* to cassava starch ratio.

## 2. Materials and Methods

### 2.1. Chemicals, Reagents, and Raw Materials

The *camote* and cassava peels were collected from a local vendor of native delicacies, and the sawdust utilized for particle board production was collected from the shavings of a local furniture shop in Vigan City, Philippines. The sawdust is typically from the shavings of mahogany, gmelina, and bamboo varieties. Borax (Sigma-Aldrich, Darmstadt, Germany), 10 wt. % sodium hypochlorite (Sigma-Aldrich, Darmstadt, Germany), 98 wt. % sodium hydroxide, hydrochloric acid (Sigma-Aldrich, Darmstadt, Germany), and 37 wt. % formaldehyde (Sigma-Aldrich, Darmstadt, Germany) were used. All methods were carried out in three trials.

### 2.2. Experimental Methodology

#### 2.2.1. Yield of Starch Extraction

For the extraction of starch from *camote* and cassava peels, 100 g of ground peels were weighed and soaked in 300 mL of lukewarm water for 60 min. The peels were removed from the container, and the starch solution was set aside for 6 h allowing the starch to settle at the bottom. The starch solution was filtered to collect the starch, and after that, the collected starch underwent moisture removal at 105 °C for 1 h in a Memmert UF30 (Schwabach, Federal Republic of Germany) oven [32]. The collected starch was weighed using an Orion HR-60 analytical balance.

#### 2.2.2. Yield of Chemically Modified Starch

The oxidation of the collected starch was carried out by preparing a forty percent starch solution (% *w*/*w*), and the pH was adjusted to 9.5 by adding drops of 2.0 M sodium hydroxide. Once a pH of 9.5 was achieved, 16 mL of sodium hypochlorite was added drop by drop with constant stirring for 30 min. The solution was stirred continuously for another 120 min, and then the pH of the solution was adjusted to 7 by adding 1 N hydrochloric acid. The solution was filtered to separate the oxidized starch from the solution [33]. The oxidized starch was dried to remove moisture and weighed using the analytical balance.

#### 2.2.3. Characterization of Adhesives in Terms of Solid Content

The next step was the preparation of the CMBWA. Five grams of oxidized starch and 50 mL of distilled water were put together in a clean beaker. The solution was warmed up at 80 °C with a constant stirring rate of 500 rpm, while 50 mL of 0.1 M sodium hydroxide and 0.2 g of borax were added. The solution was continuously heated until it started to be gelatinized.

The CMBWA was characterized in terms of its solid content and viscosity. The determination of solid content was performed by weighing 3 g of adhesive and placing it into an oven at 105 °C until a constant weight was achieved. The numerical value of the solid content was obtained using Equation (1). Where *SC* is the solid content, *w*_1_ is the initial weight of the adhesive, and *w*_2_ is the constant weight of the adhesive.

(1) $$SC\;\left(\%\right)=\frac{W_{1}-W_{2}}{W_{1}}\times 100$$

#### 2.2.4. Characterization of Adhesives in Terms of Viscosity

The viscosity was determined by filling the Zahn Series Dip Cup number 3 with CMBWA being was lifted out of the sample until all the adhesives dripped out into the Zahn Cup. The time was recorded and used to calculate the viscosity using Equation (2). Where *V* is the viscosity and *T* is the recorded time in seconds.

(2) V (Pa.s)=10.09T−587/T

#### 2.2.5. Thermal Properties of Adhesives in Terms of Glass Transition Temperature

The glass transition temperature (T_g_) was determined by applying heat at a rate of 5 °C/min to the bioadhesives. The temperature was monitored and recorded until the adhesives achieved a glassy, rubbery texture [34].

#### 2.2.6. Production and Characterization of the Particle Board in Terms of Physical Properties

For the testing of particle board produced using the CMBWA, particle board sample production was adapted from the procedure of Utleg [35], where the production of particle board samples was conducted by weighing 83 g of sawdust mixed with 125 g of adhesives. The procedure is similar to the study conducted in that the greater the amount of adhesive utilized for particle board production, the stronger the material produced [36]. The mixture was formed in a rectangular mold with a dimension of 13 cm by 13 cm by 2 cm (about 0.79 in), which is similar to the method of Ndububa [37]. The mold with the sample was compressed with a pressure of up to 20 MPa in a hydraulic press at room temperature. The particle boards were conditioned at room temperature for 24 h before their physical and mechanical testing.

The physical testing of the particle board samples was conducted in terms of water absorption based on ASTM D1037, the thickness of swelling based on ASTM D 3502-76, and density determination based on ASTM D2395. Water absorption was obtained by getting the weight of the particle board sample before and after immersing it in distilled water for 24 h and using Equation (3). Where *WA* is the water absorption and *Wo* and *Ww* are the weights of the particle board before and after immersion in distilled water for 24 h, respectively.

(3) $$WA\;\left(\%\right)=\frac{Ww-Wo}{Wo}\times 100$$

The thickness of swelling was obtained by measuring the thickness of the particle board before and after immersion in distilled water for 24 h and using Equation (4). Where *TS* is the thickness of swelling, *To* and *Tw* are the thickness of the particle board before and after immersion in distilled water, respectively.

(4) $$TS\;\left(\%\right)=\frac{Tw-To}{To}\times 100$$

The density was obtained by getting the volume and weight of the particle board samples and was computed using Equation (5). Where *D* is the density, *w* is the weight, which is expressed in grams, and *V* is the volume, which is expressed in cubic centimeters.

(5) $$D\;\left(\frac{g}{cm^{3}}\right)=\frac{w\;}{V}$$

#### 2.2.7. Characterization of the Particle Board in Terms of Mechanical Properties

The mechanical properties of the particle boards produced from CMBWA and UF adhesive were evaluated through static bending expressed using the universal testing machine model DSTM 5–25 kN in terms of modulus of rupture (*MOR*), modulus of elasticity (*MOE*), and tensile strength (*T*). The *MOR* was calculated using Equation (6). Where *F* is the force at fracture in Newton, *L* is the support span length in meters, *b* is the width in meters, and *d* is the thickness in meters. The *MOE* was calculated using Equation (7). Where stress (σ) is force divided by the specimen’s cross-sectional area and strain (ε) is the change in length of the material divided by the original gauge length of the material. The internal bond test was evaluated according to EN 319, a European standard that specifies the method for determining the tensile strength perpendicular to the plane of particleboards and fiberboards. The tensile strength and compressive strength were determined for testing the internal bond of the particle board produced. Applying Equation (8), the tensile strength was obtained, where *T* is the tensile strength, *F* is the force acting perpendicular to the particle board, and *A* is the cross-sectional area of the particle board.

(6) $$MOR\;\left(\frac{N\;}{m^{2}}\right)=\frac{3FL\;}{2bd^{2}}$$

(7) $$MOE\;\left(\frac{N\;}{m^{2}}\right)=\frac{\left(\sigma 2-\sigma 1\right)\;}{\left(\varepsilon 2-\varepsilon 1\right)}$$

(8) $$T\;\left(\frac{N\;}{m^{2}}\right)=\frac{F\;}{A}$$

#### 2.2.8. Statistical Analysis

The variability of the trial tests gathered in this study has been evaluated through statistical analysis using the standard deviation. For the obtained starch yield and other parameters tested, the mean and standard deviation with a statistical significance of 95% confidence level (*p* < 0.05) were applied. All data obtained were subjected to One-Way Statistical Analysis of Variance (ANOVA) using SPSS (version 17, 2010). Means were separated using Duncan’s Multiple Range Test (DMRT). This is carried out to determine whether samples at different camote to cassava starch ratios have different mean strengths.

## 3. Results

The following results were obtained from the extraction of starch from *camote* and cassava peels, the chemical modification of the starch extracted, and testing the particle board produced from CMBWA and comparing it with the particle board made with UF adhesives.

### 3.1. Yield of Starch Extraction

**Figure 1:**
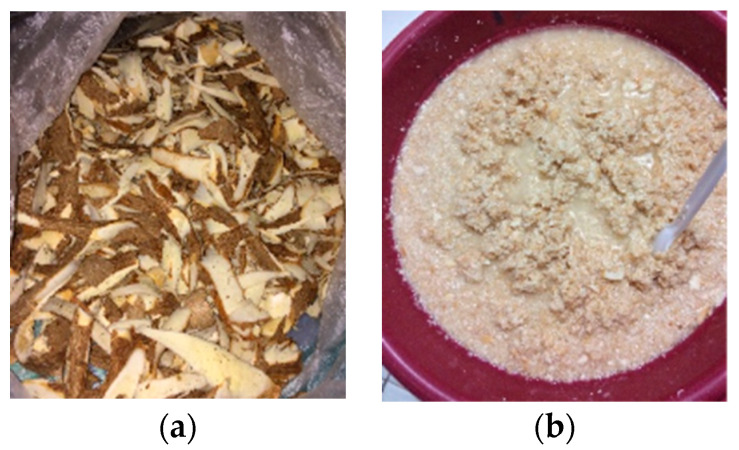
Cassava peels (**a**) utilized in this study and the actual starch (**b**) recovered from cassava peels.

Table 1 shows the percentage yield of starch extracted from camote and cassava peels, while Figure 1 shows the cassava peels utilized in this study and the actual starch recovered from cassava peels. From the 500 g of camote and cassava peels, the amount of starch extracted was 65.96 g from camote peels and 94.58 g from cassava peels. The percentage yield of starch from camote and cassava peels was 13.19 ± 0.48% and 18.92 ± 0.15%, respectively.

### 3.2. Yield of Chemically Modified Starch

From the initial mass of camote peel starch, which is 30 g, and cassava peel starch, which is 40 g before chemical modification, the value increased to 34.8 ± 1.70 g for camote and 45.34 ± 1.81 g for cassava after chemical modification, as shown in Table 2. A 16% increase for camote peel starch and a 13.35% increase for cassava peel starch are observed.

### 3.3. Characterization of Adhesives in Terms of Solid Content

Table 3 shows the solid content of the CMBWA produced. All the values exhibited almost similar solid content, but the CMBWA with a 40:60 ratio of camote and cassava starch gave the highest solid content, which is 93.6 ± 0.66%, while the CMBWA with a 20:80 ratio gave the lowest solid content, which was 92.38 ± 0.45%. The solid content for unoxidized starch adhesives is 50.92 ± 0.89%, while 95 ± 0.77% for UF adhesives.

### 3.4. Characterization of Adhesives in Terms of Viscosity

The CMBWA produced was also analyzed in terms of viscosity. Table 4 shows that the 80:20 ratio displays the lowest viscosity, which was 0.124 ± 0.01 Pa.s while the 40:60 ratio gives the highest viscosity of 0.149 ± 0.01 Pa.s. The viscosity of unoxidized starch adhesives is 0.090 ± 0.01 Pa.s, while that of UF adhesives is 0.149 ± 0.01 Pa.s.

### 3.5. Thermal Properties of Adhesives in Terms of Glass Transition Temperature

**Figure 2:**
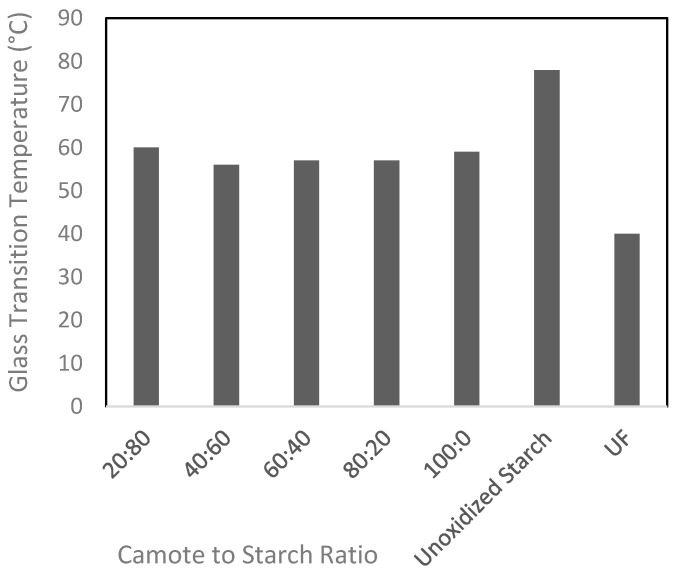
T_g_ obtained from the different camote:cassava starch ratios.

The T_g_ of the CMBWA was determined from the different camote and cassava starch ratios and is presented in Figure 2. The UF obtained the lowest T_g_ of 40 °C, while the unoxidized starch had the highest T_g_ of 78 °C. The different camote to cassava starch ratios considered for CMBWA production have T_g_ ranges from 56 °C to 60 °C.

### 3.6. Characterization of the Particle Board in Terms of Physical Properties

The production of particle boards made from sawdust using CMBWA from the different camote and cassava starch ratios was tested for its physical properties. The physical properties tested are in terms of water absorption, thickness of swelling, and density.

#### 3.6.1. Physical Properties of Particleboards in Terms of Water Absorption

Water absorption is expressed in percentages (% *w*/*w*). Table 5 shows that the 40:60 ratio gave the lowest water absorption, which is 145.03 ± 2.01%, while the 100:0 ratio gave the highest water absorption of 250.03 ± 1.08%. No value was measured for unoxidized starch because the particle board bursts and scattered, while the particle board that utilizes UF adhesives is 93.05 ± 1.16%.

#### 3.6.2. Physical Properties of Particle Boards in Terms of Thickness of Swelling

Table 6 shows that the 40:60 ratio gave the lowest thickness of swelling, which is 7.14 ± 0.32%, while the 20:80 ratio gave the highest thickness of swelling, which is 38.46 ± 0.87%. The thickness of swelling is expressed in percentages (wt. %). No value was measured for unoxidized starch because the particle board burst and scattered, while the particle board that utilizes UF adhesives is 6.03 ± 0.55%.

#### 3.6.3. Physical Properties of Particle boards in Terms of Density

Density is the measurement of mass divided by its volume. Table 7 shows that the 100:0 ratio gave the lowest density, which is 0.42 ± 0.05 g/cm^3^, while the 40:60 ratio gave the highest density of 0.48 ± 0.03 g/cm^3^. The density for unoxidized starch adhesives is 0.38 ± 0.03 g/cm^3^, while 0.71 ± 0.05 g/cm^3^ for UF adhesives.

### 3.7. Characterization of the Particle board in Terms of Mechanical Properties

The production of particle boards made from sawdust using CMBWA from the different camote and cassava starch ratios was tested for its mechanical properties in terms of MOR and MOE. Both parameters were obtained through static bending. An internal bond test was performed and evaluated by getting the tensile strength of each particle board sample.

Table 8 shows that the 60:40 ratio gave the lowest MOR and MOE, which are 3628.460 ± 29 N/m^2^ and 7.364 × 10^5^ ± 541 N/m^2^, respectively, while the 40:60 ratio gave the highest MOR and MOE, which are 6276.256 ± 118 N/m^2^ and 1.273 × 10^5^ ± 177 N/m^2^, respectively. The MOR and MOE for unoxidized starch adhesives are 3236.195 ± 59 N/m^2^ and 6.570 × 10^5^ ± 413 N/m^2^, while 13,042.845 ± 118 N/m^2^ and 2.648 × 10^6^ ± 905 N/m^2^ for UF adhesives. For the tensile and compressive strengths, 3.02 ± 0.20 and 4.20 ± 0.22 were the lowest from the camote to cassava starch ratio of 60:40, while the highest tensile and compressive strengths obtained were 5.52 ± 0.57 and 7.67 ± 0.75. The tensile and compressive strengths for unoxidized starch adhesives are 2.74 ± 0.60 and 3.80 ± 0.06, while 11.39 ± 2.11 and 15.82 ± 1.21 for UF adhesives.

## 4. Discussion

The starch from *camote* and cassava peels was extracted with a percentage yield of 13.19 ± 0.48% for *camote* and 18.92 ± 0.15% for cassava. Based on statistical analysis, the values between *camote* and cassava were far from each other. This means that there is a significant difference in the starch content on their peels. A higher percentage of starch was obtained from cassava peels than the *camote* peels because cassava naturally contains a larger amount of starch which is 25% on a wet basis and 60% on a dry basis than the *camote* with 18.56% yield [38]. The percentage yield of starch obtained in this study is 18.92 ± 0.15%, lower than the existing study, which also extracted starch from cassava peels, which is 30% [31]. The percentage yield of starch extracted from *camote* peels is 13.19 ± 0.48%. This result is still feasible since this study utilized only the peels, which have a lesser starch content than the main crop, knowing that a study conducted on extracting starch from the main crop of *camote* was able to obtain only 27–30% yield [39]. An existing study has successfully extracted and characterized starch from sweet potato peels [40]. The amount and quality of starch extracted from *camote* peels varied widely because they rely on many factors, including tuber size, maturity, variety, and even the extraction process [41].

Chemically modified starch observed an increase in mass. A 16% and 13.35% increase in mass from *camote* and cassava starch were obtained from the chemical modification, respectively. Based on statistical analysis, the values between the initial and final weights of *camote* and cassava were far from each other in the process of chemical modification. This means that there is a significant increase in weight when starch undergoes chemical modification. An increase in mass can be due to the depolymerization of starch fractions. The oxidation reaction tends to cause alleviation of the intermolecular bonds or partial depolymerization of polymer chains. The starch, when chemically modified by the oxidation process, has enhanced water solubility, a lower viscosity, and a retrograding tendency in comparison to the unoxidized one. These phenomena increased the low molecular weight of the starch fraction because of the addition of hydroxyl groups [42]. Since starch molecules contain a lot of hydroxyl groups, the substitution of these by carbonyl and carboxyl is expected during the oxidation process. These new groups make the oxidized starch obtain a higher molecular weight and its weight as well [43].

Different sources of starch have diverse attributes such as viscosity, gelatinization temperature, and adhesive strength [44]. The solid content of adhesives influences its brushability, adhesive tack time, and bonding strength because an increase in the solid content of the adhesive reduces its water content [45]. For adhesives, the standard solid content is 55–57%, but the higher the solid content of the adhesives, the better [46]. All the *camote* and cassava starch ratios formulated have excellent solid content values. Adhesive properties improved with increasing solid content and a lower solid content indicates that more volatiles need to be removed during the pressing process [47]. The improvement of the solid content is caused by the oxidation of the starch, which introduces carboxyl and carbonyl groups that cause the adhesive formulated to retard recrystallization [48]. Oxidized starch increases stability against excessive heat, acid, shear, time, cooling, and freezing [49]. Based on statistical analysis, the values within the five *camote* to cassava starch ratios were very close to each other and can be regarded as having no significant difference. In adhesives, the higher the solid content, the better. When it comes to drying time, the best ratio will be the one that obtains the highest solid content value.

Another property of the CMBWA to be considered is its viscosity. The lower the viscosity of the binder, the better the liquidity will be. All the formulations considered obtain a low viscosity profile, but the *camote* to cassava ratio of 80:20 observes the lowest viscosity among them. The liquidity of the adhesive affects the permeability of the adhesive and the uniformity of the dispensing shot range. It also affects the production speed, board bonding performance, planeness, and strength of boards [50]. A reduction in viscosity was noticed on the CMBWA compared to the viscosity of unmodified starch. The probable reason is due to the breakdown of linkages in the starch during the oxidation process. According to Mohamaddi et al. [51], another likely reason for the reduced viscosity was due to the extensive disruption of the amorphous region in starch granules and conversion to a low molecular weight chain. Based on statistical analysis, the values within the five *camote* to cassava starch ratios were close to each other, and there was no significant difference between their values. In adhesives, the higher the viscosity, the better adhesive property; moreover, the best ratio will be the one that obtains the highest viscosity. The viscosity of 40:60 is like the viscosity of UF in the control sample.

It is also important to evaluate adhesives in terms of their thermal properties. One important parameter for thermal properties is T_g_. It is a critical temperature for the effectiveness of adhesives and other polymer materials. T_g_ is the temperature in reference to polymer transitions from a rigid, glassy state to a more flexible, rubbery state [1]. In the case of adhesives, T_g_ is essential due to its influences on their mechanical properties, such as flexibility, toughness, and adhesion strength [52]. T_g_ is a significant parameter to consider in adhesive applications because it can affect the performance of the adhesive under different temperature conditions. Adhesives with a T_g_ below the expected service temperature may become too soft and lose their bonding strength, while adhesives with a Tg above the service temperature may become too brittle. Below the T_g_, the adhesive is typically in a glassy or brittle state. In this condition, the ability of the adhesive to deform and absorb energy is limited. Above T_g_, the adhesive becomes more pliable and can absorb mechanical stresses [53]. Therefore, adhesives with lower T_g_ are more advantageous than those with higher T_g_. In the study, the data show that chemical modification of starch adhesives improves their T_g_, as indicated by the lower T_g_ for CMBWA compared to the unoxidized starch adhesives. The CMBWA produced did not undergo further characterization of its morphology. However, in a study where the characterization of modified starch was conducted through Scanning Electron Microscope (SEM), it was found that the water resistance of starch adhesive was significantly improved by chemical modification [54].

The bioadhesive produced was then applied for particle board production and tested in terms of water absorption, thickness of swelling, density, MOR, MOE, and tensile strength. The water absorption determines how much water the particle board constructed using the CMBWA has absorbed after subjecting it to specified conditions. The maximum limit of water absorption is 40%. However, all the samples exceeded this limit. The result of this study is opposite from the existing study conducted, where oxidization of starch improved the water resistance property of the particle board constructed from it [45]. One of the possible reasons that affected the water absorption of the particle board is the pressing method, wherein the particleboard side was not smoothed properly, which caused the penetration of water into the material. Penetration of water on the particle board might also be the crystalline disruption that has occurred during the oxidation of the *camote* and cassava peel starch. The crystalline structure of the starch tends to be due to the interaction of hydrogen bonding between starch and water. Oxidization of starch improves the water resistance of the adhesives due to the elimination of hydroxyl groups in the starch as it undergoes oxidation [50]. Based on statistical analysis through ANOVA, the values within the five *camote* to cassava starch ratios were very far from each other and can be regarded as having a significant difference. The interaction between the wood particles and the type and proportion of starch adhesives during the application process can vary, thereby influencing the water absorption characteristics of the product. Combining different sources can create a formulation that takes advantage of the strengths of each source, producing a more versatile and efficient adhesive [55]. In a comparison study conducted where different sources of starch were utilized as a source of adhesive for particle board production, it was found that a significant difference in the mechanical properties of particle board was observed among the different sources of starch [56]. In particle boards, the lower the water absorption, the better; the best ratio will be the one that obtains the lowest water absorption value.

The thickness of the swelling of the particle boards constructed was also tested. For the particle board produced and constructed using CMBWA, the ratio of 40:60 gave the best result with a value of 7.14 ± 0.32%. The standard for the thickness of swelling in particle boards is a maximum of 15% after a 24 h immersion in water [57]. The thickness of swelling for the produced particle boards from CMBWA was within the standard. This result indicates that chemical modification improved the bonding strength of the starch utilized for wood adhesive [45]. The result of this study is also like the study conducted where the oxidization of starch was used for the formulation of adhesives for plywood construction, wherein the bonding strength of the starch was improved because of the oxidation process [58]. The anisotropic swelling response is also due to the materials utilized for particle board production. Soft wood materials, although they absorb less moisture, would result in thicker swelling behavior [59]. The sawdust that was collected for particle board production came from different wood varieties. There is a tendency that the other adhesive ratios considered were made from softer wood material than the 20:80 ratio. Based on statistical analysis, the values within the five *camote* to cassava starch ratios were very far from each other except for 60:40, 80:20, and 100:0, where no significant difference was observed. The 20:80, 40:60, and the other three *camote* to cassava starch ratios have significant differences, which means that a remarkable difference is observed. In terms of thickness of swelling, the lower the thickness of swelling, the better when it comes to the quality of particle board. Therefore, the best ratio will be the one that obtains the lowest thickness of swelling.

Density is said to be a measure of the compactness of the individual particles on the board. The density of particleboard produced from CMBWA was tested. For the particle board produced from a 40:60 ratio of *camote* to cassava, the density was 0.48 ± 0.03 g/cm^3^. The standard density for particleboard for LD-1 and LD-2 is less than 0.64 g/cm^3^ [60]. Concerning density, the produced particleboards can be classified as LD-1 and LD-2, which means the particleboard produced can be used for door core [61]. Based on statistical analysis, the values within the five *camote* to cassava starch ratios were very close to each other, which means that no significant difference was observed between the values considered. In terms of density, when it comes to particle board, the higher the density, the better, so the best ratio will be the one that obtains the highest density.

The compressive strength of a particle board is influenced by the reinforcement and resin in the particle board. The common drawback under compressive force in a particle board is the twining of load-bearing reinforcement. The greater the compressive strength, the better the particle board produces [62]. Compressive strength is directly proportional to MOR and MOE, which means that an increase in compressive strength would also result in an increase in MOR and MOE. MOR and MOE of the different CMBWA ratios show that all are inferior compared to the set standards for MOR and MOE. The compacting parameters were not met during the pressing of the particleboard, particularly the pressing temperature. However, among the CMBWA ratios considered, the 40:60 ratio obtained the best mechanical properties in terms of compressive strength and MOR. In the study conducted, it was found out that pressing temperature affects the mechanical properties of particle board [63]. Based on statistical analysis, the five ratios have no significant difference in terms of tensile strength. The 60:40 ratio shows a significant difference in terms of compressive strength, as shown in Table 8, while the rest of the *camote* to cassava starch ratios have no significant differences from each other. However, the values within the five *camote*:cassava starch ratios were far from each other and can be regarded as a significant difference being observed for MOE and MOR. Data shows that MOR, MOE, and tensile strength are directly proportional to each other. Different *camote*:cassava starch adhesive ratios obtain different values in terms of mechanical properties because various sources and proportions of starch as adhesives exhibit unique properties. An investigation of the mechanical properties of particle boards made from different sources of starch as bioadhesives revealed that a significant difference is obvious [30]. The composition of the adhesive, viscosity, and bonding characteristics play a role in determining its effectiveness in binding wood particles. Consequently, these factors impact the overall tensile strength of the particle board. In terms of MOR and MOE in particle board, the higher the MOR and MOE, the better, so the best ratio will be the one that obtains the highest MOR and MOE. For the internal bond test, the tensile strength is directly proportional to the compressive strength. Similar results have been obtained by the study that tested particle boards produced using Gum Arabic resin as adhesives [37].

## 5. Conclusions

In the manufacturing of *camote* and cassava CMBWA, the process of starch modification enhanced the properties of the starch intended for wood adhesives. The extraction of starch from *camote* and cassava peels yielded approximately 13.19 ± 0.48% and 18.92 ± 0.15%, respectively. A significant improvement is observed among all the parameters tested for oxidized starch utilized for wood adhesives compared to unoxidized starch. A starch ratio of 40:60 demonstrated the most favorable combination for solid content and viscosity. When CMBWA was applied in particleboard production, the 40:60 starch ratio showed superior results in terms of compressive strength and modulus of rupture. Additionally, this ratio yielded the best outcomes for density, thickness, swelling, and water absorption. According to the study findings, the characteristics of CMBWA, particularly the solid content, surpassed established standards. Overall, the 40:60 *camote* to cassava starch ratio proved to be the most effective among the different *camote* to starch ratios considered since, among the parameters tested, this ratio obtained most of the best properties. However, it is worth noting that when CMBWA was employed in particleboard production, some of the tested parameters did not meet the required standards. The resulting particleboards can be categorized as LD-1 and LD-2 grade, indicating their suitability for applications such as door cores. To enhance the physical and mechanical properties, further investigation into the impact of pressing temperature on particleboard construction using CMBWA is recommended. Another step to sufficiently support and prove that the starch extracted from cassava and *camote* peels is effective as a bio-based wood adhesive is further characterization through Nuclear Magnetic Resonance, Scanning Electron Microscopy, thermogravimetric analysis, and Fourier-transform infrared spectroscopy. Analyzing the morphology of materials and other spectroscopical aspects is also an excellent step to further enhance this study. Digital Scanning Calorimetry is also recommended to support the data obtained for T_g_. Identifying and conducting particle size distribution on the sawdust to be utilized for particle board production as part of testing the CMBWA is also important for the improvement of the study. Investigating the potential of the material produced for sound and thermal insulation is also recommended to widen its utilization. Utilizing locally available raw materials would lessen the dependence on imported adhesive. It can also lessen pollution since the waste *camote* and cassava peels which are usually burned for disposal are converted into useful products. Using these raw materials can help eliminate the dependence on synthetic wood adhesives which are non-renewable. This study, if materialized, would be a significant help to the people in the community because starch from waste *camote* and cassava peels, once converted into wood adhesive could be a source of additional income.

## Figures and Tables

**Table 1:** Yield of starch extraction.

Samples	Mean Starch Yield (g)	Mean Starch Yield (%)
*Camote*	65.96 ± 1.143 ^b^	13.19 ± 0.48 ^b^
Cassava	94.58 ± 0.543 ^a^	18.92 ± 0.15 ^a^

Values are means (*n* = 3) ± standard deviation; means with different superscripts (a,b) within the same column denote that there is a statistically significant difference (*p* < 0.05).

**Table 2:** Yield of chemically modified starch.

Samples	Initial Mass (g)	Mean Mass of Chemically Modified Starch (g)
*Camote*	30	34.80 ± 1.70 ^b^
Cassava	40	45.34 ± 1.81 ^a^

Values are means (*n* = 3) ± standard deviation; means with different superscripts (a,b) within the same column denote that there is a statistically significant difference (*p* < 0.05).

**Table 3:** Solids content of CMBWA.

Ratio of *Camote* to Cassava Starch	Solid Content (%)
20:80	92.38 ± 0.45 ^a^
40:60	93.60 ± 0.66 ^a^
60:40	92.56 ± 0.33 ^a^
80:20	92.85 ± 0.63 ^a^
100:0	92.66 ± 0.41 ^a^
Unoxidized starch	50.92 ± 0.89
UF	95.00 ± 0.77

Values are means (*n* = 3) ± standard deviation; means with different superscripts within the same column denote that there is a statistically significant difference (*p* < 0.05).

**Table 4:** CMBWA in terms of viscosity.

Ratio of *Camote* to Cassava Starch	Viscosity (Pa.s)
20:80	0.139 ± 0.13 ^a^
40:60	0.149 ± 0.01 ^a^
60:40	0.147 ± 0.01 ^a^
80:20	0.124 ± 0.01 ^a^
100:0	0.140 ± 0.01 ^a^
Unoxidized starch	0.090 ± 0.01
UF	0.149 ± 0.01

Values are means (*n* = 3) ± standard deviation; means with different superscripts within the same column denote that there is a statistically significant difference (*p* < 0.05).

**Table 5:** Particle boards in terms of water absorption.

Ratio of *Camote* to Cassava Starch	Water Absorption (%)
20:80	180.75 ± 1.18 ^c^
40:60	145.03 ± 2.01 ^e^
60:40	178.57 ± 1.32 ^d^
80:20	243.63 ± 1.89 ^b^
100:0	250.03 ± 1.08 ^a^
Unoxidized starch	---
UF	93.05 ± 1.16

Values are means (*n* = 3) ± standard deviation; means with different superscripts (a–e) within the same column denote that there is a statistically significant difference (*p* < 0.05).

**Table 6:** Particle boards in terms of thickness of swelling.

Ratio of *Camote* to Cassava Starch	Thickness of Swelling (%)
20:80	38.46 ± 0.87 ^a^
40:60	7.14 ± 0.32 ^c^
60:40	13.33 ± 1.08 ^b^
80:20	11.47 ± 0.40 ^b^
100:0	14.29 ± 0.99 ^b^
Unoxidized starch	---
UF	6.03 ± 0.55

Values are means (*n* = 3) ± standard deviation; means with different superscripts (a–c) within the same column denote that there is a statistically significant difference (*p* < 0.05).

**Table 7:** Particle boards in terms of density.

Ratio of *Camote* to Cassava Starch	Density (g/cm^3^)
20:80	0.44 ± 0.04 ^a^
40:60	0.48 ± 0.03 ^a^
60:40	0.45 ± 0.03 ^a^
80:20	0.43 ± 0.04 ^a^
100:0	0.42 ± 0.05 ^a^
Unoxidized starch	0.38 ± 0.03
UF	0.71 ± 0.05

Values are means (*n* = 3) ± standard deviation; means with different superscripts within the same column denote that there is a statistically significant difference (*p* < 0.05).

**Table 8:** Mechanical properties of particleboards.

Ratio of *Camote* to Cassava Starch	MOR (N/m^2^)	MOE (N/m^2^)	Tensile Strength (N/m^2^)	Compressive Strength (N/m^2^)
20:80	5687.857 ± 128 ^b^	1.154 × 10^5^ ± 324 ^b^	4.97 ± 0.10 ^a^	6.90 ± 0.09 ^a^
40:60	6276.256 ± 118 ^a^	1.273 × 10^5^ ± 177 ^a^	5.52 ± 0.57 ^a^	7.67 ± 0.75 ^a^
60:40	3628.460 ± 29 ^e^	7.364 × 10^5^ ± 541 ^e^	3.02 ± 0.20 ^a^	4.20 ± 0.22 ^b^
80:20	4118.793 ± 59 ^d^	8.365 × 10^5^ ± 285 ^d^	3.48 ± 0.31 ^a^	4.84 ± 0.13 ^a^
100:0	5491.724 ± 59 ^c^	1.115 × 10^5^ ± 393 ^c^	5.05 ± 0.17 ^a^	7.01 ± 0.10 ^a^
Unoxidized starch	3236.195 ± 59	6.570 × 10^5^ ± 413	2.74 ± 0.60	3.80 ± 0.06
UF	13,042.845 ± 118	2.648 × 10^6^ ± 905	11.39 ± 2.11	15.82 ± 1.21

Values are means (*n* = 3) ± standard deviation; means with different superscripts (a–e) within the same column denote that there is a statistically significant difference (*p* < 0.05).

## Data Availability

The data presented in this study are available on request from the corresponding author.
